# MRI-Guided Focused Ultrasound in Parkinson's Disease: A Review

**DOI:** 10.1155/2017/8124624

**Published:** 2017-03-30

**Authors:** Ilana Schlesinger, Alon Sinai, Menashe Zaaroor

**Affiliations:** ^1^Department of Neurology, Rambam Health Care Campus, Haifa, Israel; ^2^Technion Faculty of Medicine, Haifa, Israel; ^3^Department of Neurosurgery, Rambam Health Care Campus, Haifa, Israel

## Abstract

MRI-guided focused ultrasound is a new technology that enables intracranial ablation. Since lesioning ameliorates some of the symptoms of PD, this technology is being explored as a possible treatment for medication resistant symptoms in PD patients. The purpose of this paper is to review the clinical use and treatment outcomes of PD patients treated to date with this technology.

## 1. Introduction

Parkinson's disease (PD) is a neurodegenerative disorder characterized by tremor, rigidity, and bradykinesia. Tremor is the most common initial symptom of Parkinson's disease (PD), observed in about 50% of patients at the time of diagnosis [1–3]. The classic PD tremor is the rest tremor that has its greatest amplitude with the limb at rest and transiently disappears with movement but can reemerge with sustained posture. Postural/kinetic tremor is seen in a subset of patients with PD. The pathophysiology of tremor may be different from other PD symptoms which may explain why tremor responds less well to dopaminergic treatments [4]. Tremor-dominant PD is characterized by prominent tremor of one or more limbs with relative bradykinesia and rigidity. The tremor-dominant patients are not only disabled by the tremor itself but also suffer social isolation that further decreases quality of life [5]. A wide range of medications have been used to treat tremor including levodopa, dopamine agonists, anticholinergics, botulinum toxin, clozapine, amantadine, clonazepam, propranolol, and neurontin, but some PD patients have disabling tremor that is refractory to medications. In these patients, neurosurgical intervention is a treatment option.

Another unmet need in PD is treatment of motor fluctuations and dyskinesia that occur as a complication of levodopa therapy [6]. Since all patients with PD will eventually receive levodopa, treating this complication is crucial. Motor complications usually develop within 5 to 10 years of starting this medication [7]. Motor complications are fluctuations between effective control of symptoms, “ON” state, and reemergence of the PD symptoms, “OFF” state. These fluctuating symptoms may be accompanied by involuntary movement called dyskinesias. Though catechol-O-methyltransferase inhibitors [8], dopamine agonists, amantadine, and MAO-B inhibitors improve symptoms in some patients, their effectiveness is limited by declining tolerability with disease progression while complimentary medicine offers only short-term relief of symptoms [9]. Before the introduction of levodopa treatment, thalamotomy and pallidotomy were the mainstay of treatment [10]. Today, neurosurgical intervention, most commonly deep brain stimulation, is proposed, but, though this is an effective treatment, many patients are reluctant to undergo this procedure because of the need to undergo burr holes with insertion of foreign bodies, intracerebral electrodes, and an implanted pulse generator [11–13]. Gamma knife stereotactic radiosurgery is also an option; although effective, it may have delayed serious adverse events [14, 15].

MRI-guided focused ultrasound (MRgFUS) is a new technology that enables intracranial ablation. Since lesioning ameliorates some of the symptoms of PD [16], this technology is being explored as a possible treatment for medication resistant symptoms in PD patients. It has already been approved for the treatment of essential tremor. The purpose of this paper is to review the clinical use and treatment outcomes of PD patients treated with this technology.

## 2. MRI-Guided Focused Ultrasound

The first to suggest using focused ultrasound beams to treat movement disorders were Fry et al. [17]. In 1958, they reported utilizing four focused ultrasound beams to treat numerous human patients suffering from various movement disorders, in particular Parkinson's disease [18]. In order to focus the beams to the specific brain region at high intensity, they performed craniotomies which eliminate absorption of the rays by the bone, which caused heating of the skull [18, 19]. Today, technological advances allow focusing high intensity ultrasound beams through an intact skull without incisions or burr holes [20, 21]. Advances in MRI technology allow accurate targeting and real-time thermal monitoring using MR thermometry [22], thus providing accurate localization and control of the thermal dose needed for effective ablation [23].

In the MRgFUS procedure, a small brain target is heated with ultrasound rays, a technique called sonication. With this technique, the MRI serves as the surgeon's eyes for targeting the energy and the US rays serve as the surgeon's knife for creating the lesion. These rays heat the tissue, thereby causing thalamotomy since heating any tissue normal or abnormal to 57°C for one second (or an equivalent thermal dose) denatures protein, thus causing 100% cell death. The area of tissue exposed to the temperature and the length of exposure to this heat defines an equivalent thermal dose [24] which determines the extent of the lesion. By focusing the heat to more than one point or by scanning the focus, a volume of tissue can be thermally ablated.

MRgFUS surgery is performed in the MRI suit. The MRI is used for target definition, treatment planning, and intervention guidance with high precision. Simultaneous real-time monitoring of the temperature at the target is achieved with MR thermometry [23], allowing for a gradual procedure that enables lesion effect monitoring at clinically low temperatures when the effects and possible side effects are still presumed to be reversible. Definitive nonreversible thermal ablation is performed only after temporary tremor reduction when the patient reports no adverse effect.

MRgFUS ablation of the thalamus for the relief of tremor was performed by us [25, 26] in a stepwise fashion using ExAblate 2000 (InSightec Inc., Haifa, Israel). In the first stage, sonication is delivered at very low energy to confirm that the sonication focus is in the selected target. This is verified in 3 planes (sagittal, axial, and coronal). When needed, the sonication focus is adjusted. The temperature at this stage typically reaches 41–46°C. The second stage involves sonication with gradually increasing energy to achieve a temporary effect on tremor and to confirm the absence of adverse effects. The target is continuously examined for accuracy according to the planned coordinates and to the patient's clinical and neurological examination including tremor evaluation by drawing spirals, writing, cup holding, and other preoperative known tremor triggering maneuvers. The coordinates are repositioned when necessary according to the clinical status, the patients' feedback, and adverse effects, if any. When no amelioration of tremor is seen, the sonication focus is moved until tremor reduction is achieved taking into account the anatomy of the VIM somatotopic arrangement and its thalamic surrounding nuclei [27, 28]. Typically, temperature at this stage reaches 46–50°C. The third stage, the ablation stage, includes a gradual increase in total energy by either increasing intensity of sonication or by prolonging sonication duration. Sonication is stopped when adequate control of tremor is achieved, with the temperature reaching no more than 60°C. It is possible to repeat the sonication with the maximal energy to verify long lasting effect.

Pallidothalamic tractotomy was performed by Magara et al. [29] in a similar fashion except that at ablative temperatures they first performed a single sonication; but after they concluded that the effect of a single sonication did not persist, they performed multiple sonication with better results, as described below. The exact technique of pallidotomy using MRgFUS has yet to be described in the literature.

A subset of patients have skull features that do not allow enough heating of intracranial structures. This can usually be foreseen by performing a brain CT scan before treatment and calculating a skull density ratio [30]. Low skull density may preclude treatment.

## 3. MRI-Guided Focused Ultrasound Targets

### 3.1. Pallidothalamic Tractotomy

The first to report treating PD patients with MRgFUS were Magara et al. [29]. They chose to perform pallidothalamic tractotomy, a target rarely approached with previous lesioning methods. Their rationale was that, in the 1960s, subthalamic lesioning showed symptom relief in PD patients and a histological study they performed led them to believe they were aiming at the thalamic fasciculus. With this target, they reported treating 13 patients (Table 1). They selected patients with refractory symptoms, 9 with tremor predominant PD that experienced severe tremor and 4 with akinetic rigidity with motor fluctuations. In the first four patients, they performed a single sonication at the high temperature which causes ablation. In these patients, beneficial effect did not last and tremor returned within 3 months. Therefore, in the next 9 patients, they performed repeated sonication and reported good results. The validated tool for measuring changes in function, the Unified PD Rating Scale (UPDRS), is reported as the outcome measure, though these authors used a self-modified, not validated, version of the scale. They chose to score mentation, behavior, mood, activity of daily living, and motor complications according to the original UPDRS. But in the motor part of the UPDRS (part III), they declared that they scored only the treated side of the body to a maximal score of 56, while, in the original scale, bilateral signs as well as axial signs are scored to a maximal score of 108. In doing this, they scored axial and bilateral symptoms but did not score axial and bilateral signs. They also do not report whether the UPDRS was performed in the ON or OFF state. They report a reduction of UPDRS-motor from 18.8 ± 11.7 at baseline to 16.5 ± 8.2 (2.3 points, 12% relief) at three months after a single sonication at ablative temperature in 4 patients. In 9 patients, they report a reduction in the UPDRS-motor score from 18.7 ± 5.2 at baseline to 7.3 ± 4.5 (11.3 points, 61% relief) at three months *p* < 0.001 after repeated sonication (Table 2). The global symptom relief was reduced by 22.5% and 56.7%, respectively. They reported no adverse event during or after sonication.

The lesion they performed was on target with a mean 3D vector accuracy of 1.0 mm. Lesion volumes for the patients with lasting results was on average of 172 mm^3^ in comparison with 83 mm^3^ with a single sonication. The temperature at the target on the last sonication ranged between 52°C and 59°C (average 56.2°C). The mean sonication duration was 13 s (range 10–21 s), the maximum acoustic power was 1,200 W, and the maximum applied energy was 20,400 J. They report no target hemorrhages or other untoward tissue reactions.

### 3.2. Ventral Intermediate Nucleus Thalamotomy

We reported in 2015 our experience with ventral intermediate nucleus thalamotomy (VIM) in 7 medication resistant, tremor predominant PD patients. The VIM was calculated to be located anterior to the posterior commissure, at 25% of the distance from the anterior to the posterior commissure, 14 mm lateral to the anterior-posterior commissural line. In cases where the 3rd ventricle was widened, the initial target was 11.5 mm lateral to the III ventricular wall. In 2016, we reported our experience in 5 additional PD patients [25, 26] (Table 1). Of these 12 PD patients, five received levodopa with motor fluctuations. Tremor stopped in the treated hand in all patients immediately following the treatment. Three patients had essential tremor for many years before they developed PD. UPDRS was examined in the ON state. At one month, UPDRS-motor part decreased from 24.9 ± 8.0 before treatment to 16.4 ± 11.1 (8.5 points, 34.1% relief) after MRgFUS (*p* = 0.042). UPDRS continued to improve over time and was 13.4 ± 9.2 (11.5 points, 46.2% relief) at six months (*p* = 0.009, as compared with baseline) (Table 2). At one month, item 20 and item 21 of the UPDRS (rest and action tremor) decreased from 2.9 ± 1.0 to 0.4 ± 1.0 (*p* < 0.001) and from 3.00 ± 1.2 to 0.6 ± 1.0, respectively, and, at 6 months, to 0.3 ± 0.5 and 0.6 ± 1.1, respectively. PDQ39, a validated quality of life questionnaire used in PD patients, decreased from 38.6 ± 16.8 before treatment to 26.1 ± 7.2 at one month posttreatment (*p* = 0.036) This measure also continued to improve over time and at six months scores reached 20.6 ± 8.8 (*p* = 0.008, as compared with baseline). During follow-up of 6–24 months (mean 11.6 ± 7.2 months, median 12.0 months), tremor reappeared in four of the patients. Two PD patients that suffered from essential tremor for many years before they developed PD were among those had tremor recurrence. In these patients, both rest and kinetic tremor recurred.

Adverse events that transiently occurred during sonication in PD patients included the following: short lasting vertigo (*n* = 5), headache (*n* = 4), dizziness (*n* = 3), nausea (*n* = 1), burning scalp sensation (*n* = 1), and lip paresthesia (*n* = 1). Adverse events that lasted after the procedure included the following: asthenia (*n* = 2), gait ataxia (*n* = 1), unsteady feeling (*n* = 1), unilateral taste disturbances (*n* = 1), and hand ataxia (*n* = 1). No adverse event lasted beyond 3 months. For a complete list of adverse events reported in this paper which also included essential tremor patients, see [26].

Lesions in the planned target were close to spherical with a diameter of 4–9 mm (average, 6.8 ± 1.5 mm). We observed mild edema one day after the procedure with increased edema one week after the procedure. The edema lasted for 3–5 weeks following the procedure. At 3 months, the edema resolved and lesion decreased in size.

The mean maximal temperature at the target was 56.5 ± 2.2°C (range, 55–60°C). Patients underwent on average 21.1 ± 8.3 sonication (range, 14–45) with an average maximal sonication time of 16.2 ± 3.0 seconds (range, 13–24). The mean maximal energy reached was 12,750 ± 4385 Joules (range, 5850–23040).

Recently, Bond et al. [31] reported in abstract form preliminary results of their double-blinded, randomized controlled trial to investigate the effectiveness of MRgFUS thalamotomy in tremor-dominant PD. They found that MRgFUS showed a trend toward improvement in hand tremor and a clinically significant reduction in mean UPDRS. They also noted a significant placebo response. Further information regarding this study is not yet available.

### 3.3. Pallidotomy

There is one report by Na et al. of lesioning of the globus pallidus interna using MRgFUS [32] (Table 1). They reported their experience in a single woman with severe levodopa in disabling motor fluctuations and severe levodopa-related motor complications. Total UPDRS was 53 at baseline and was reduced to 16 at one month, 14 at 3 months, and 18 at 6 months (37, 39, and 35 points, 69.8%, 73.5%, and 66.0% relief, respectively). The UPDRS-motor part improved from 10 at baseline to 5 at 3 months (Table 2). On MRI, the lesion was distinct one month after treatment and was seen less clearly at 3 months. They did not report adverse events.

On imaging, they observed signal changes consistent with pallidotomy and degree of signal changes was decreased at 3 months.

## 4. Discussion

MRgFUS is a new emerging treatment for medications resistant symptoms in PD. To date, patients underwent this treatment in order to relieve medication resistant tremor and for disabling motor complications. Significant benefit was seen in most patients with very few transient adverse events.

The target chosen for MRgFUS differed according to the treatment center. Magara et al. chose to lesion the pallidothalamic tract to relieve tremor and dyskinesia [29]. We chose to perform thalamotomy for PD patients with severe tremor [25, 26]. While Na et al. chose to perform pallidotomy in order to ameliorate motor complication [32]. It is not clear which target is most beneficial and whether different targets should be chosen for different symptoms. What makes it hard to compare the targets is the use of different treatment outcome measures. For instance, we used the original motor UPDRS (part III) [25, 26] as our main outcome measure, while Magara et al. [29] used a self-modified UPDRS-motor score, making the scores not comparable. Interestingly, the improvement in the UPDRS-motor scores reported by Magara and by us shows similar improvement (11.3 points in Magara's paper and 11.5 points in our paper), suggesting similar improvement in both treatment targets. More information regarding efficacy and adverse events will be needed in order to answer this question.

Long-term efficacy is also an important issue. Magara et al. reported that, in order to avoid symptom recurrence, repeated ablation at high temperatures at treatment target is needed [29]. This repeated sonication resulted in larger lesions which may account for the better long-term outcome. We reported the longest follow-up of PD patients that underwent MRgFUS [26]. In our experience, 4 of 12 patients had some tremor recurrence. We noted that tremor recurrence was more frequent in patients that suffered from essential tremor for many years before they developed PD. Though we treated only 3 such patients, tremor recurred in 2 of them. This was in comparison to tremor recurrence in 2 of 9 patients with PD with no previous neurological deficit. This observation suggests that MRgFUS may not be the optimal choice in this unique group of patients of PD following essential tremor.

Pallidotomy is a promising target for MRgFUS, but there are unique challenges in treating this target. The first is the technical issue of focusing ultrasound rays to this target because they are at the edge of the treatment area. The second is finding the exact target within the pallidum that will ameliorate symptoms. The third is the proximity of the optic nerve to the globus pallidus internus. To date, it is not clear what will be the best target for treating PD symptoms or whether different targets should be used for different patients. Another question that has yet to be answered is whether bilateral treatments can be performed since currently all treatments are unilateral in order to avoid possibly serious adverse events. Adverse events recently reported in a multicenter trial of focused ultrasound thalamotomy for essential tremor, where most centers had little experience with this technology, included gait disturbances and paresthesias or numbness that persisted after 12 months. Therefore, maybe focused ultrasound thalamotomy should be performed in specialized centers with experience in the procedure [33].

MRgFUS is a new option for PD patients with medication resistant symptoms. It is approved for this indication in Israel, Europe, Korea, and Russia. Further studies are needed in order to better characterize patient selection and treatment targets.

## Figures and Tables

**Table 1:** Characteristics of Parkinson's disease patients that underwent MRgFUS treatment.

Authors & year	Target	Patients, *n*	Age, y (range)	Sex, m	Tremor dominant	Levodopa treatment	Disease duration, y (range)
Magara et al. 2014	Pallidothalamic tract	13	64.5 (37–82)	8	9	11	9.7 (3–27)
Schlesinger et al. 2015	Thalamotomy	7	59 (46–74)	6	7	3	5.4 (2.5–10)
Na et al. 2015	Pallidotomy	1	56	0	0	1	12
Zaaroor et al. 2017	Thalamotomy	12^*∗*^	62.8 (46–75)	11	12	5	7.1 (2–16)

^*∗*^Of the 12 patients in Zaaroor et al., 7 are the same patients as in Schlesinger et al.

**Table 2:** Treatment outcomes of Parkinson's disease patients that underwent MRgFUS treatment.

Authors & year	Target	Patients, *n*	Motor UPDRS (Part II)				
			Pretreatment	Post treatment 3 months	Point reduction	Change (%)	Significance
Magara et al. 2014 Group 1	Pallidothalamic tract	4	18.8^*∗*^	16.5	2.3	12.2	NA
Magara et al. 2014 Group 2	Pallidothalamic tract	9	18.7^*∗*^	7.3	11.4	61.0	*p* < 0.001
Schlesinger et al. 2015	Thalamotomy	7	27.0	19.7	7.3	27.0	*p* = 0.036
Na et al. 2015	Pallidotomy	1	10	5	5	50	NA
Zaaroor et al. 2017	Thalamotomy	12^*∗∗*^	24.9	13.4^*∗∗∗*^	11.5	46.2	*p* = 0.002

^*∗*^A modified, not validated version of the motor-UPDRS that scored only the treated side of the body to a maximal score of 56 instead of 108 points in the original scale.

^*∗∗*^Of the 12 patients in Zaaroor et al., 7 are the same patients as in Schlesinger et al.

^*∗∗∗*^UPDRS at 6 months.
